# Evaluating healthcare providers’ knowledge and attitudes towards disaster management policies in a Johannesburg hospital

**DOI:** 10.4102/curationis.v49i1.2847

**Published:** 2026-06-24

**Authors:** Muladelo Godzwana, Emmanuel E.O. Agbenyeku

**Affiliations:** 1 Department of Environmental Health, Faculty of Health Science, University of Johannesburg, Johannesburg, South Africa; 2 Department of Construction Management and Quantity Surveying, Faculty of Engineering, University of Johannesburg, Johannesburg, South Africa; 3 Department of Civil Engineering Technology, Faculty of Engineering, University of Johannesburg, Johannesburg, South Africa

**Keywords:** disaster, preparedness, disaster management, policy, health care providers, knowledge, attitude

## Abstract

**Background:**

Disasters in hospitals, such as epidemics, fires and flooding, are a global concern, especially in South Africa. Many healthcare providers lack knowledge of disaster management and preparedness policies (DMPP).

**Objectives:**

To assess the knowledge and attitudes of Healthcare Providers (HCPs) regarding DMPP in a selected hospital in Johannesburg, to determine whether the knowledge and attitudes of HCPs regarding DMPP are related to training and to determine whether the knowledge, attitudes and training of HCPs regarding DMPP differ according to their type of work and level of education.

**Methods:**

A quantitative cross-sectional descriptive design was applied in the study. Analysis of variance and multilinear regression analysis were utilised to find the relationships between knowledge, attitudes and training.

**Results:**

The study had an adequate response rate of 86. Results showed that enrolled nurse assistants had better knowledge and attitudes about DMPP than other healthcare providers.

**Conclusion:**

The study concluded that many HCPs still need improvement in knowledge and attitudes regarding DMPP and recommends creating training programmes and better collaboration amongst health and safety committees for effective disaster management.

**Contribution:**

The study provides a significant perspective about knowledge and attitudes of HCPs in DMPP, it identifies gaps in awareness, training and implementation of disaster protocols, therefore providing evidence to guide policy improvement, targeted disaster education and HCPs capacity-building initiatives to strengthen hospital disaster readiness in South Africa, in essence enhancing National Health Insurance readiness.

## Introduction

Disasters are one of the unexpected disruptive events that affect hospitals worldwide. The most common disasters affecting the hospital setting in the Republic of South Africa (RSA) are epidemics leading to sudden surges of patients, fires, flooding and bomb threats (Barten et al. 2021:2). Such disasters can cause massive damage and disruption of healthcare services (Shabbir et al. 2017:464). Over the past 5 years in South Africa, more than 10 hospitals have had fires and flooding in their units. In a media press article by Wilson (2022:1), it is reported that since January 2020, 17 hospitals across the country have experienced fires, and whilst many were extinguished quickly and with minimal damage done.

These fires outbreak specially highlight the lack of maintenance and failing infrastructure in South African health facilities. However, the knowledge and attitude of healthcare providers towards disaster management and preparedness policies (DMPPs) are crucial. Similarly, Khirekar et al. (2023:3) and Goniewicz et al. (2023:8) outlined that various prevention, mitigation and reduction strategies, such as training, drills, daily disaster and emergency monitoring and role assignments, are used for disaster preparedness in hospitals.

Rahman (2022:1) reported that, globally, cases of different disasters were recorded to be 432 in 2021, which accounted for 10.492 deaths worldwide, inclusive of healthcare users, emergency personnel and healthcare providers. According to the United Nations Office for Disaster Risk Reduction 2021 report (UNDRR 2021:28), Sub-Saharan Africa had over 100 disaster cases, which affected the functionality of healthcare facilities, disrupted care provision and even led to the deaths of patients. According to Times Live (2022), the estimated value of more than 1.1 billion rands was lost on hospital property because of an unforeseen fire outbreak in South African hospitals.

Disaster management and preparedness policy is enacted by the National Disaster Framework and the National Disaster Management Act, Act 57 of 2002. The framework is developed so that all government spheres, including healthcare providers, are knowledgeable about preparing for unforeseen events. Drills and training in disaster management are aspects that are emphasised by the DMPP frameworks. Guo et al. (2025:4) reiterate in their study that drills and training enhance the knowledge and attitudes towards disaster management and preparedness activities. Phukubye, Mbombi and Mothiba (2019:444) discovered that amongst the nurses with knowledge of triaging, 61% exercised good triage practice, whilst only 39% showed evidence of poor practice. The balance is crucial for effective disaster preparedness; moreover, triage, amongst other activities, is the prescript emphasised by the National Disaster Frameworks Act 57 of 2002. The assessment of the knowledge and attitude of healthcare providers on DMPP in South Africa is a critical aspect of public health and emergency response.

Disaster management and preparedness policies are essential for ensuring that healthcare providers are equipped to respond effectively to natural disasters, pandemics and other emergencies (Herstein et al. 2021:1). Furthermore, understanding the knowledge and attitudes of healthcare providers towards DMPP is crucial for identifying gaps in training, resources and support. The training of healthcare professionals in disaster and emergency preparedness skills is used as a strategy to prevent the intense disruptions of disasters, as per findings by Naser and Saleem (2018:2).

The National Core Standards (NCS) are used to monitor and evaluate compliance of the health facilities in South Africa regarding disaster preparedness (National Department of Health 2011). This is based on the NCS for Health Establishments version 2011, developed by the RSA National Department of Health. The NCS are also supported by the regulations relating to Norms and Standards under the National Health Act, Act 61 of 2003. The National Health Act stipulates that health facilities must follow the rules for being prepared for disasters. The Office of Health Standards Compliance (OHSC) is a quality assurance health entity that monitors and evaluates compliance to mandate of NCS in different domains, including disaster preparedness and compliance.

The NCS are important for health facilities to follow, so they can be ready for disasters. Naser and Saleem (2018:2) further elaborate that insufficient knowledge of healthcare professionals on DMPP contributes to ineffective disaster management and preparedness practices. Several studies focused on practice and compliance, rather than determining the HCPs’ knowledge and attitude on DMPP. The study aims to assess the knowledge and attitudes of HCPs regarding DMPP in a selected hospital in Johannesburg,

## Research methods and design

### Study design

A quantitative, cross-sectional descriptive research was used to study healthcare providers, namely doctors, psychologists, pharmacists, enrolled nurses, enrolled nurse assistants (ENAs), professional nurses, social workers, occupational therapists, radiographers and physiotherapists regarding their knowledge and attitude of DMPP.

### Study settings

The study was conducted in different units of one academic hospital in Johannesburg, Gauteng region. The study setting had approximately 1500 health providers employed and comprised approximately 3400 beds.

### Study population and sampling strategy

The study population included healthcare providers with different levels of healthcare qualifications such as doctors, professional nurses, enrolled nurses, ENAs, social workers, occupational therapists, physiotherapists, radiographers, pharmacists and psychologists.

Sample size was estimated using EPIINFO version 7.2.4.0, which had a Cochrane’s formula. A 95% of confidence interval (CI) and an error margin of 5% and 50% of distribution rate were calculated based on the 1500 HCPs in the selected hospital. A sample size of 383 was derived after adding a contingency of 25%. Nonprobability purposive sampling was used to select samples from the targeted population of doctors, professional nurses, enrolled nurses, ENAs, social workers, occupational therapists, physiotherapists, radiographers, pharmacists, psychologists and others. HCPs retrieved were ENAs (16.4%), enrolled nurses (15.8%), professional nurses (7.0%), physiotherapists (11.2%), occupational therapists (7.6%), radiographers (10.3%), psychologists (4.2%), medical doctors (26.1%) and 1.5% specifying other roles.

### Data collection

Data were collected using a structured, closed-ended questionnaire designed in English. The questionnaire was self-developed with statistician, and internal consistency was validated using Cronbach’s alpha and factor analysis, and 0.95 reliability was obtained. The questionnaires were divided into four sections (demographics, attitudes, knowledge and training in disaster management policy). To explore the knowledge and attitude regarding DMPP, Likert scales were used. The 383 hard copies of questionnaires were physically distributed to different unit managers’ offices. Three hundred and thirty questionnaires were retrieved from tamper-sealed boxes between August 2023 and September 2023; therefore, 86.2% of participants responded.

### Analysis

Data were captured on an excel worksheet and imported to Statistical Package for Social Sciences (SPSS) version 28.0 for analysis. Missing values and data entry errors were identified and corrected by list-wise deletion. Descriptive statistics were performed to understand the distribution of sociodemographic factors of the study. Frequency and percentage were obtained for the categorical variables. The knowledge and attitude of healthcare providers were measured, and independent variables such as training, years of experience and type of occupation were described. A one-way Analysis of Variance (ANOVA) was conducted to compare the effect of training, knowledge and attitude amongst various occupations, level of education and work experience regarding disaster management and the preparedness of the policy. Lastly, a multiple linear regression analysis was used to predict knowledge, attitude and training status based on sociodemographic indicators. A *p*-value of < 0.05 was a cut-off for statistically significant measures, with 95% CI.

### Ethical consideration

Ethical approval to conduct the analysis was obtained from the University of Johannesburg Human Research Ethics Committee (REC-2087-2023). Permission to conduct this research study was obtained from the Hospital Research Ethics Committee and the Head of Medical, Surgical and Allied healthcare department. Research information sheet, which outlined the aims, ethical consideration and risks and benefits, was distributed to participants in different hospital units. Written informed consent of participants was obtained in different hospital units prior to data collection. The privacy and confidentiality of the respondents were protected as there was no attempt to identify the subjects’ names in the data. Thus, all data used in this study were anonymised.

## Results

### Demographic data

A total of 330 healthcare workers responded to the questionnaires. The gender distribution was nearly equal with 48.8% male, 50.9% female and 0.3% prefer not to disclose. The largest age group was 36–45 years (68.5%), followed by 26–35 years (20.6%), whilst 9.4% were aged 46 years and older, and 1.5% were 18–25 years. In relation to education, most participants held a bachelor’s degree (60.0%), followed by national diplomas (25.8%), higher certificates (10.0%) and master’s degrees (4.2%). Occupation categories for healthcare workers include medical doctors (26.1%), nurse assistants enrolled (16.4%), enrolled nurses (15.8%), physiotherapists (11.2%), radiographers (10.3%), occupational therapists (7.6%), professional nurses (7.0%), psychologists (4.2%) and other specified roles (1.5%). Regarding work experience, most participants (59.7%) had ≥ 7 years of experience, 33.3% reported 5–6 years, 5.5% reported 3–4 years and 1.5% reported 1–2 years. No participant reported less than 1 year of work experience (Table 1).

**TABLE 1:** Sociodemographic characteristics of healthcare workers in a selected hospital in Johannesburg (*N* = 330).

Variable	Category	Frequency (*n*)	%
Gender	Male	161	48.8
	Female	168	50.9
	Prefer not to say	1	0.3
Age (years)	18–25	5	1.5
	26–35	68	20.6
	36–45	226	68.5
	≥ 46	31	9.4
Education level	Higher certificate	33	10.0
	National diploma	85	25.8
	Bachelor’s degree	198	60.0
	Master’s degree	14	4.2
Occupation	Enrolled nurse assistant	54	16.4
	Enrolled nurse	52	15.8
	Professional nurse	23	7.0
	Physiotherapist	37	11.2
	Occupational therapist	25	7.6
	Radiographer	34	10.3
	Psychologist	14	4.2
	Medical doctor	86	26.1
	Other, specify	5	1.5
Work experience	Below a year	0	0.0
	1–2 years	5	1.5
	3–4 years	18	5.5
	5–6 years	110	33.3
	7+ years	197	59.7

### Training status on disaster management and preparedness policy

In South Africa, DMPP measures revealed a distinct pattern across the three domains (training, knowledge and attitude status). Training status demonstrated the lowest mean score (*M* = 1.55, standard deviation [s.d.] = 0.79) with a positively skewed distribution (skewness = 1.58) that indicates the majority of respondents had limited training experience; these findings are constant with those of Susila et al. (2019), who observed that fewer that 30% of healthcare providers in the institutions are exposed to disaster preparedness training, therefore highlighting significant limitation in capacity building. In contrast, knowledge status showed the highest mean score (*M* = 3.80, s.d. = 0.54) with a negatively skewed distribution (skewness = −1.97), which suggests that most participants possessed relatively higher knowledge levels. Attitude status yielded an intermediate mean score (*M* = 2.31, s.d. = 1.13) with an approximately normal distribution (skewness = 0.26, kurtosis = −1.26) that indicated a balanced spread of attitudes towards DMPP amongst respondents (Table 2).

**TABLE 2:** Descriptive statistics for disaster management and preparedness policy measures amongst healthcare workers (*N* = 330).

Measure	Training status	Knowledge status	Attitude status
Mean	1.55	3.80	2.31
Standard deviation	0.79	0.54	1.13

### Group differences in disaster management and preparedness policy measures

One-way ANOVA analysis showed that there was statistically significant differences across occupational groups for training (*F*[8321] = 14.96, *p* < 0.001), knowledge (*F*[8321] = 4.832, *p* < 0.001) and attitude (*F*[8321] = 26.748, *p* < 0.001), as well as across education levels for training (*F*[3326] = 6.385, *p* < 0.001), knowledge (*F*[3326] = 15.849, *p* < 0.001) and attitude (*F*[3326] = 7.224, *p* < 0.001) in relation to DMPP.

The post hoc analysis for occupational groups showed that the ENAs scored higher in training than physiotherapists and medical doctors’ category, but lower than occupational therapists. For knowledge, ENAs scored significantly lower than the medical doctors’ category, whereas enrolled nurses demonstrated superior knowledge compared to professional nurses, physiotherapists and the medical doctors’ category.

Attitude status revealed that ENAs showed lower scores than enrolled nurses and the medical doctors’ category, whilst enrolled nurses exhibited significantly more positive attitudes than most other occupational groups. Educational level analyses revealed that participants with national diplomas demonstrated higher training scores than bachelor’s degree holders, those with higher certificates showed superior knowledge compared to diploma holders but diploma holders outperformed master’s degree holders and healthcare workers with higher certificates displayed less positive attitudes than both diploma and bachelor’s degree holders (*p* = 0.005), though diploma holders maintained more positive attitudes than master’s degree holders. In relation to work experience, the analysis showed that work experience did not significantly influence training status (*F*[3326] = 2.224, *p* = 0.085), knowledge levels (*F*[3326] = 1.929, *p* = 0.125) or attitudes (*F*[3326] = 1.553, *p* = 0.201) regarding DMPP (Table 3).

**TABLE 3:** One-way ANOVA results for training, knowledge and attitude by occupation, education level and work experience amongst healthcare workers (*N* = 330).

Components	*F*-statistic	*df*	*p*-value
**Occupational**	-	**8321**	-
Training	14.96	-	< 0.001
Knowledge	4.83	-	< 0.001
Attitude	26.75	-	< 0.001
**Education level**	-	**3326**	-
Training	6.39	-	< 0.001
Knowledge	15.85	-	< 0.001
Attitude	7.22	-	< 0.001
**Work experience**	-	**3326**	-
Training	2.22	-	0.085
Knowledge	1.93	-	0.125
Attitude	1.55	-	0.20

*df*, degree of freedom.

### Predicators of disaster management and preparedness policy

Multiple linear regression analyses were conducted to examine the predictive relationships between sociodemographic factors and disaster management preparedness measures amongst healthcare workers. The models revealed varying explanatory power, with training status showing the highest variance explained (adjusted *R*^2^ = 0.129), followed by attitude status (adjusted *R*^2^ = 0.093) and knowledge status (adjusted *R*^2^ = 0.073). For training status, significant predictors included gender (β = 0.67, *t* = 2.52, *p* = 0.012), education level (β = 0.22, *t* = 2.49, *p* = 0.013), job type (β = −0.14, *t* = −5.91, *p* < 0.001) and work experience (β = 0.17, *t* = 2.43, *p* = 0.016), with male gender, higher education and greater work experience associated with higher training scores, whilst certain job types were associated with lower training scores. Attitude status was significantly predicted by age (β = 0.45, *t* = 3.92, *p* < 0.001), education level (β = −0.36, *t* = −2.85, *p* = 0.005) and job type (β = 0.15, *t* = 4.25, *p* < 0.001), indicating that older age and specific job categories were associated with more positive attitudes, whilst higher education was linked to less positive attitudes. Knowledge status showed the most limited predictive model, with only job type emerging as a significant predictor (β = 0.07, *t* = 4.06, *p* < 0.001), suggesting that certain occupational roles were associated with higher knowledge scores, whilst other sociodemographic factors had minimal influence on knowledge levels (Table 4).

**TABLE 4:** Multiple linear regression coefficients for predicting disaster management and preparedness policy measures by sociodemographic factors.

Variable	Training status			Attitude status			Knowledge status		
	β	*t*-value	*p*-value	β	*t*-value	*p*-value	β	*t*-value	*p*-value
Gender	0.67	2.52	0.012	−0.20	−1.75	0.082	−0.11	−1.89	0.060
Level of education	0.22	2.49	0.013	−0.36	−2.85	0.005	−0.06	−1.04	0.298
Job type	−0.14	−5.91	< 0.001	0.15	4.25	< 0.001	0.07	4.06	< 0.001
Work experience	0.17	2.43	0.016	−0.18	−1.78	0.076	0.05	1.08	0.282
Age	−0.03	−0.41	0.380	0.45	3.92	< 0.001	−0.05	−0.85	0.399
Adjusted *R*^2^	-	0.129	-	-	0.093	-	-	0.073	-

## Discussion

This study contributes to the understanding of disaster management and preparedness amongst healthcare workers in South Africa, highlighting challenges such as limited training, uneven knowledge levels and variations in attitudes across professional categories. The findings inform the development of a structured framework for strengthening disaster preparedness within healthcare facilities. Drawing on empirical evidence and lessons from policy resilience, the proposed approach emphasises systematic training, knowledge enhancement and attitudinal change as critical components for improving readiness to respond to disaster-related risks. In the study, a mean knowledge status of 3.80 with a s.d. of 0.54 was found by analysing the knowledge status distribution. The data were found to be negatively skewed (−1.97), which indicates that a significant proportion of healthcare workers are low in knowledge status, whilst a smaller number of healthcare workers have high knowledge status. Regarding attitude, some healthcare providers have differing attitudes towards DMPPs: Negative attitude (95%) and positive attitude (5%). These results support previous literature of Naser and Saleem (2018:4) who found in the study that few healthcare providers still had good attitudes towards disaster management and preparedness, amongst the 23.2% who received training.

Also, in the study, findings imply that advanced age (36 years to ≥ 46) is linked to a poor attitude towards DMPP. Contrarily, Labrague et al. (2018:50) argue that age was not a factor associated with knowledge of DMPP. The studies suggest that women may have poorer knowledge regarding DMPP. This implies that gender has a small significance in contributing to either negative or poor attitudes towards DMPP. Gaillard et al. (2017:866) findings support the notion that gender dimensions are an insufficient construct with which to address knowledge and attitude towards disaster management and preparedness.

In clarification, a national diploma qualification is awarded to ENAs and professional nurses only, whilst a bachelor’s degree is awarded to doctors and other allied healthcare workers. These results make sense because categories of nurses are more exposed to disaster management and preparedness training and workshops than other healthcare providers. There is no previous study supporting that assistant nurses have a poor attitude; however, Basal and Ahmed (2018:151) support that 62.14% of highly educated registered nurses have a positive attitude towards DMPP. These findings support the idea that attitudes differ according to the level of education. These results make sense because mostly the enrolled nurses are given tasks to ensure safety in the wards and daily checking of disaster management and preparedness aspects, such as fire extinguishers. Shanableh et al. (2023:1) support that medical doctors have a more positive attitude towards disaster management and preparedness as compared to other job types of healthcare providers. However, previous studies found that work experience plays a crucial role in shaping the knowledge and attitude of healthcare providers towards DMPP. Walles et al. (2023:310) and Ahayalimudin and Osman (2016:1) support that healthcare providers with more than 5 years of working experience are more likely to have a positive attitude and sufficient knowledge towards disaster and emergency preparedness than participants with less than 5 years’ work experience.

### Strengths and limitations

The study has public health implications, such as emphasis on implementing policies to address a lack of knowledge and a poor attitude towards disaster management and preparedness amongst HCPs. The study has added knowledge to the existing evidence-based research in disaster management and preparedness. Limitations, such as using a cross-sectional design, was conducted over a period of 2 months, and some participants resigned during the process of data collection. The study did not have responses from speech therapists, dental therapists or dentists; this might have contributed to power differences and the inability to reach the expected sample size. In addition, this study only focused on one tertiary academic hospital in Johannesburg; therefore, it would be inappropriate to generalise the findings to other academic hospitals in Gauteng province.

### Recommendations

The study recommends that not only training of HCPs on DMPP is essential, but also periodically well-grounded disaster exercises that resemble actual events to enhance the preparedness of healthcare providers are pivotal. Continuous monitoring and evaluation of compliance are essential to support and enforce attitudes towards disaster preparedness. The establishment of a disaster committee in each unit is essential for effective disaster management and preparedness.

## Conclusion

There is an increasing worldwide concern regarding disasters and their consequences. This study was performed to identify the current levels of knowledge and attitude of healthcare providers regarding DMPP in a selected hospital. Lack of knowledge amongst healthcare providers regarding DMPPs is still a concern in public tertiary and academic hospitals. This is reflected in the current study and previous studies on disaster management and preparedness. Enrolled nurse assistants reported having a high level of knowledge as compared to other health categories. In South Africa, it was observed through clinical exposure in public health facilities that ENAs are tasked with daily checking of disaster compliance in the ward, that is, checking fire extinguishers, emergency trolley checklists and alarming systems, which may be the reason for a high level of knowledge. Poor knowledge and varied attitudes regarding DMPPs amongst healthcare providers need to be addressed. Henceforth, improving the knowledge and attitudes of healthcare providers in DMPP is essential for enhancing the overall resilience of healthcare systems in responding to disasters, especially during the times of National Health Insurance (NHI) phase implementation. Without resilience, various disasters can disrupt access to service delivery, health economy, health infrastructures or even lead to high mortality rates, thereby undermining progress towards universal health coverage envisaged by NHI. A resilient system enhances continuity of care, efficient resource use and the ability to respond and recover quickly, thereby supporting both disaster preparedness and management and the successful implementation of NHI.
